# The Family and Medical Leave Act: A Policy Analysis and Recommendations to Address Employed Caregiver Burden

**DOI:** 10.1093/geroni/igaa057.344

**Published:** 2020-12-16

**Authors:** Geunhye Park, Erin Robinson

**Affiliations:** University of Missouri-Columbia, Columbia, Missouri, United States

## Abstract

Family caregiving plays a pivotal role in the long-term care system in the U.S, as there are over 40.4 million people providing unpaid care to individuals aged 65+ (U.S. Bureau of Labor Statistics, 2019). The majority are women providing supports to a parent/grandparent and provide an average of three hours of care each day. This places greater demands on family caregivers in balancing their dual caregiver/employment roles. The Family and Medical Leave Act (FMLA) of 1993 enables family caregivers to take unpaid leave to provide supports to immediate family. While FMLA was intended to provide flexibility to employed caregivers, many struggle with family-work conflicts and caregiver burden is high. Therefore, this conceptual paper offers a critical examination of FMLA and how family caregivers of older adults are impacted. Results of this analysis revealed three themes. First, FMLA is largely inadequate for employed caregivers, as only 60% of the workforce are eligible and unpaid leave restrictions create considerable financial hardship. Second, employer discrimination is high and family caregiving discrimination claims have dramatically increased since FMLA was enacted. And third, many employed caregivers are unaware of FMLA policies and eligibility requirements, which results in underutilization of benefits. Based upon these results, several policy and employer recommendations can be made, such as expanding FMLA coverage to include paid leave and non-immediate family caregivers. Additional recommendations will also be addressed. As it has been nearly 30 years since FMLA was enacted, updated policy is vital to continue supporting employed caregivers in their roles.

